# MAD saccade: statistically robust saccade threshold estimation via the median absolute deviation

**DOI:** 10.16910/jemr.12.8.3

**Published:** 2020-05-12

**Authors:** Benjamin Voloh, Marcus R. Watson, Seth König, Thilo Womelsdorf

**Affiliations:** Vanderbilt University Nashville, USA; York University Toronto, Canada

**Keywords:** Saccades, median absolute deviation, MAD, eye tracking, head-free viewing

## Abstract

Saccade detection is a critical step in the analysis of gaze data. A common method for saccade detection is to use a simple threshold for velocity or acceleration values, which can be estimated from the data using the mean and standard deviation. However, this method has the downside of being influenced by the very signal it is trying to detect, the outlying velocities or accelerations that occur during saccades. We propose instead to use the median absolute deviation (MAD), a robust estimator of dispersion that is not influenced by outliers. We modify an algorithm proposed by Nyström and colleagues, and quantify saccade detection performance in both simulated and human data. Our modified algorithm shows a significant and marked improvement in saccade detection - showing both more true positives and less false negatives – especially under higher noise levels. We conclude that robust estimators can be widely adopted in other common, automatic gaze classification algorithms due to their ease of implementation.

## Introduction

The analysis of gaze behaviour yields critical insights into processes underlying visual attention, perception, and executive control, as well as their mechanistic underpinnings (1, 2, 3). One prominent component of gaze behaviour is saccades, ballistic eye movements that rapidly re-orient the eye and thus the image impinging on the retina. Saccades typically last between 20 and 100 ms, depending on the amplitude of the saccade (1, 4, 5). Because of their ballistic nature, saccades can be differentiated from other gaze events – such as fixations or smooth pursuits – by examining the eye velocity and/or acceleration (6, 7, 8, 9, 10, 11). A common approach is to apply a threshold in the velocity (e.g., Nyström & Holmqvist, 2010) and/or acceleration domain (e.g., Duchowski et al., 2002), on the basis of the known physiology of eye movements. Threshold crossings mark the presence of saccades, and further analysis can then demarcate more precise onsets and offsets (10, 11). Although other, computationally more elaborate methods exist that may outperform thresholding algorithms under certain conditions (9, 12, 13, 14, 15), threshold algorithms have the advantage that they are relatively simple to implement, and are effective in experiments with rigorously defined, simple gaze behaviour.

A critical and common step in algorithmic saccade detection is thus the choice of the threshold. However, variability in saccadic profiles, the presence of other gaze events such as fixations or smooth pursuits (10), measurement noise (16, 17), or sampling frequency (18,19), all make it difficult to reliably detect saccades algorithmically. Indeed, in the presence of these factors, event detection critically depends on the choice of threshold (5, 14, 20, 21). To circumvent this problem, an alternative approach is to estimate a threshold from the data itself, which may adapt to changing conditions (2, 11, 22,23).

**Figure 1.**
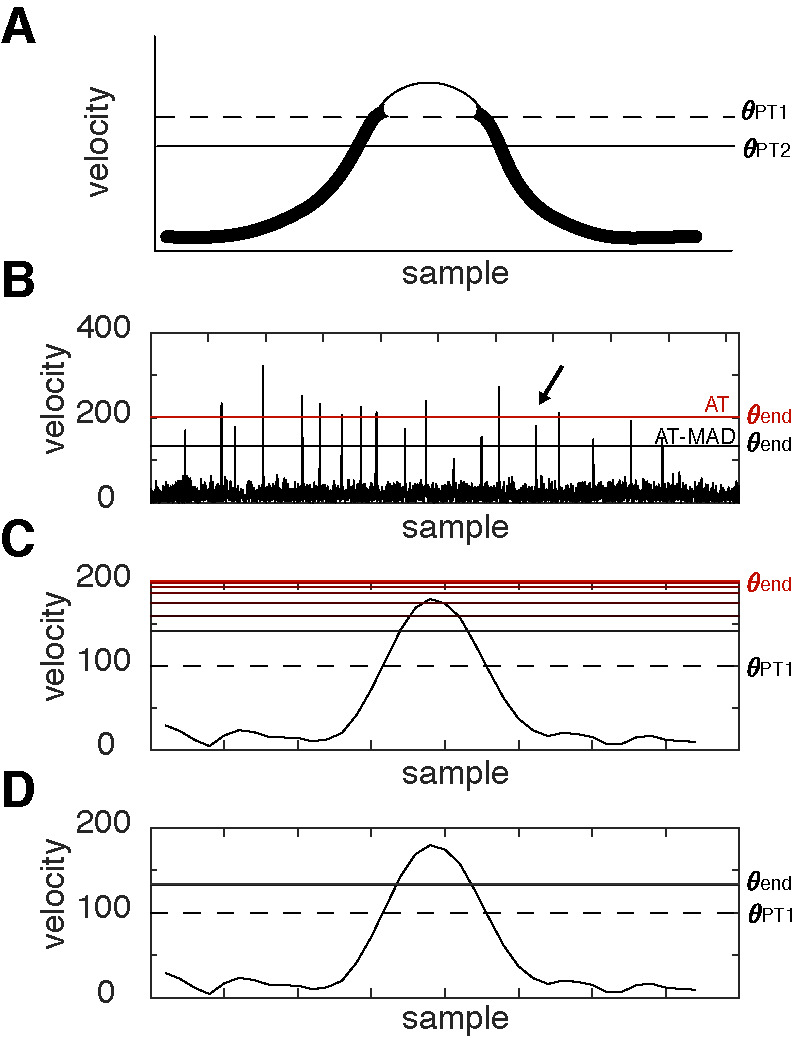
Example where adaptive thresholding detects saccades in a data-driven manner, but can fail with non-robust statistics **(A)** Schematic of AT algorithm. The threshold for detection is initialized at θ_PT1 _(dashed line). All points below this (thick line segments of the curve) are then used to calculate θ_PT2 _(solid horizontal line), a new threshold used on the next iteration to determine the next threshold This algorithm proceeds until it converges to a solution. **(B)** Velocity of simulated scanpath with 20 low amplitude saccades. Horizontal lines depict the final threshold θ_end_ as determined by the AT algorithm (red) and AT-MAD algorithm (black). The AT-MAD algorithm finds a lower bound than AT, though still well above the background noise. (**C-D) **Example saccade that was not detected by the AT algorithm (C) but was detected by the AT-MAD algorithm (D), corresponding to the arrow in (B). (see Figure 5E-J for examples from human data). The initial threshold is depicted as a dashed line. Solid, red horizontal lines represent the threshold on successive iterations, with darker (lighter) lines showing earlier (later) iterations. Notice that it increases beyond the initial threshold, but the AT-MAD algorithm successfully stops iterating, whereas AT does not.

Recently, Nyström and colleagues developed an algorithm that iteratively calculates an adaptive velocity threshold, which has the benefit of setting the lowest possible threshold given changes in background noise and fixation characteristics (11). This algorithm performs better than nine other recent algorithms on data measured while participants viewed static stimuli (12). It iteratively calculates the threshold as a function of the mean and standard deviation. However, because these quantities are highly biased by the presence of outliers, the outliers may be undetected, a phenomenon called "masking” (24). For a given sampling frequency, saccades are by definition outliers in the velocity or acceleration domain, as they take up a far smaller number of gaze points than fixational intervals, and they have much higher peak velocities and accelerations. This implies that the saccade detection threshold is modulated by the very signal it is trying to detect (Figure 1). A robust estimation of the saccade threshold would ideally be independent of saccades.

The purpose of this work is to formally compare saccade detection using statistically robust and non-robust threshold estimation. We have further set out two goals for threshold estimation; first, it should be robust and insensitive to variations of maximum saccade amplitudes; and second, that it can be efficiently implemented. To this end, we modify the algorithm by Nyström and colleagues by using the median and median absolute deviation (MAD), which are robust to outliers in the data (24, 25). The median absolute deviation is a robust estimate of dispersion, that, when properly scaled, can estimate the standard deviation of different distributions (24, 25). Previous gaze research has used this measure to detect outliers and clean data (26), while other research has recommended using a median filtering to reduce the influence of noise in general (27, 28). We propose using MAD as a threshold estimator in and of itself. We find that robust threshold estimation leads to improved saccade detection, particularly at higher noise levels, and is robust to varying threshold confidence levels. It also improves the performance of the original algorithm as proposed by Nyström and colleagues. We conclude that threshold estimation based on MAD can be widely and easily applied in other saccade detection algorithms where the threshold should be robustly estimated (e.g., Larsson et al., 2013).

## Methods

All analyses were performed in MATLAB 2015b (Mathworks) using custom code. Implementation code for the robust estimation and saccade simulation is available at: https://github.com/att-circ-contrl/mad_saccade

We first provide a brief overview of the study. We motivate our study by simulating saccades of varying amplitudes and under different levels of noise. We then modify an existing saccade detection adaptive-threshold algorithm using robust statistics, and go on to show how our modified algorithm performs under different levels of noise and choices of free parameters. We further validate our results in human participants performing a task under head-free viewing conditions.

### Saccade Simulation

We simulated saccades to create ground truth scan paths in order to objectively compare algorithms. Simulations were based on the procedure proposed by (16). Two dimensional saccades were generated using a parametric model of saccades that reproduces the saccadic main sequence. The parametric model generates a saccade waveform from the sum of a soft ramp function and a shifted negated soft ramp function (Equation 2. (16)). We used the following parameter values for Equation 2 selected from uniform distributions ranging between these listed values : η= 0.45-0.65, c = 4.5-7.5, and τ = 2-6. Importantly, τ represents saccade amplitude. We used saccade amplitudes ranging from 2-6 degrees of visual angle (dva), representing ranges that are prevalent in experiments with static images in humans. Noise-free scanpaths were generated at a sampling rate of 500 Hz. Ground-truth onset/offsets for each saccade were defined as the first point where the velocity dropped below 5 deg/s in the noise-free simulation. Measurement noise was simulated by adding white noise (standard deviation range, [0 1]).

### Adaptive Algorithm for Saccade Detection

Nyström and colleagues (2010) proposed an innovative method to determine the saccade threshold in an adaptive, iterative way (Figure 1A) ((11); *see also* (8) for a complementary description). It first determines a global velocity threshold for saccade detection on the basis of putative fixational periods, and then local, flanking velocity subthresholds that are the basis for onset/offset estimation. We obtained code for the adaptive algorithm from the personal website of Marcus Nyström ("http://www.humlab.lu.se/en/person/MarcusNystrom/", link: “*Source code for the algorithm described in Nyström, M., & Holmqvist, K. (2010). An adaptive algorithm for fixation, saccade, and glissade detection in eyetracking data. Behavior research methods, 42(1), 188-204.”*). This code was slightly modified from its published version to allow for parameter testing. We refer interested readers to the original publication for the full details of the algorithm, but describe here the relevant details for the adaptive threshold calculation. 

Velocities were calculated via the Savitsky-Galoy filter (order=2, span=40 ms) (11). The determination of the saccade peak velocity threshold θ_PT_ can be broken down into the following steps (Figure 1A). First, θ_PT_ is set to an initial value in the range 100-300 deg/s. Second, for all velocity samples *x* lower than θ_PT_, a new threshold is calculated as:

**(1):**
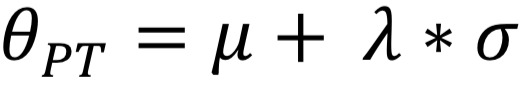


where μ and σ is the mean and standard deviation over samples *x,* and the parameter λ (lambda) is a scale factor equal to 6. This procedure is then repeated until the error between iterations is less than 1 deg/s. The block of samples above ${𝜃}_{\mathrm{PT}}$ are a putative saccade. To determine saccade onsets, the algorithm first looks back in time from the putative saccade to the first point that crosses the saccade onset threshold ${𝜃}_{\mathrm{ST}}^{\mathrm{onset}}$:

**(2):**
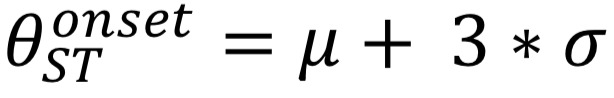


If this threshold is crossed, the algorithm continues back in time to the nearest local velocity minimum, which is defined as the saccade onset. The procedure is similar for saccade offsets, with the exception that the saccade offset threshold ${𝜃}_{\mathrm{ST}}^{\mathrm{offset}}$ is defined as:

**(3):**
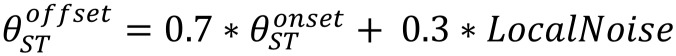


Where *LocalNoise* was defined as the mean and 3 times the standard deviation of the velocity signals in the 40 ms preceding the saccade start. The saccade offset was defined as a local minimum after the last crossing of ${𝜃}_{\mathrm{ST}}^{\mathrm{offset}}$ (see (11) for details).

In our tests, we compare two versions of this original algorithm. First, we use the algorithm as presented on the website. However, in the current implementation (1.0), the algorithm does not (re-)calculate the threshold over all remaining data samples at each iteration. Instead, for each putative inter-saccadic interval (i.e. between threshold crossings), a number of samples are removed at the start and end of the inter-saccadic interval, defined as the minimum fixation duration (40 ms) * sampling frequency (500 Hz) / 6, which comes out to 3 samples removed at the flanks of each inter-saccadic intervals. In our simulations, this amounts to the removal of ~1% of the data. Thus, a second version of the algorithm does *not* excise any parts of the data, which is the algorithm as originally proposed in the publication. We refer to the first version – the *adaptive threshold* algorithm with excised data - as “AT-excise”, and the second one using all data as “AT”.

### Robust estimation of mean and deviation

To get a robust estimate of the central tendency and variability of the data, we instead propose to use the median and median absolute deviation (MAD) (24, 25) . In this framework, we treat saccades as outliers to be detected (2) . A robust measure of variability σ’ is defined as:

**(4):**
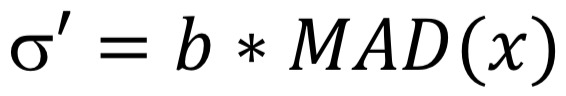


**(5):**
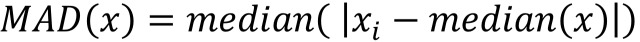


MAD on its own tends to underestimate the standard deviation, and thus must be scaled by the factor *b*. The factor *b* is equal to 1.4826 assuming the underlying distribution (i.e. ignoring outliers) is normal (24, 25) . The distribution of velocities can differ based on the type of gaze behaviour (4, 8), and this can be accounted for by setting b to the inverse of the 75 ^th^ percentile (24, 25). Thus, we proposed to calculate the peak velocity and saccade onset/offset thresholds as:

**(6):**
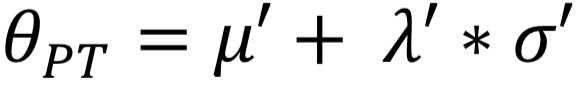


**(7):**
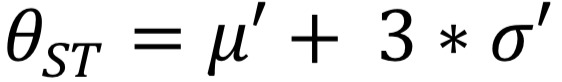


where μ’ is the median. We refer to this algorithm as “AT-MAD”, and also compare it to one where we excise data (as described above), referred to as “AT-MAD-excise”. We will use the term “robust” to refer to algorithms using robust statistics (AT-MAD and AT-MAD-excise). 

Previous research has suggested that adaptive thresholds perform better than fixed threshold under certain conditions (2, 11, 16), though a recent study has shown the inverse to be true (8). To this end, we also compared our modified algorithm to a fixed threshold version, where the velocity threshold was set at 55 deg/sec and the saccade onset/offset threshold was set at 45 deg/sec. (8).

### Algorithm Comparison

To [XMLmind] the performance of different algorithms, we used event-based comparison to match true and detected saccades (16, 29) . True saccades were extracted from simulated, or later, manually annotated experimental data (see below). A match is logged if there is sufficient sample overlap (>20%) between a true and detected saccade (16, 29). All matched saccades are true positives (TP), unmatched true saccades are false negatives (FN), and unmatched detected saccades are false positives (FP). From these, we further calculate the precision (=TP/TP+FP) and recall (=TP/(TP+FN)) (16, 29). These are used to compute the F1 score (=2*precision*recall/(precision+recall)), an aggregate performance measure.

To determine the timing characteristics of onsets and offsets, we take their difference between the (matched) true and detected saccades. Onset/offset lags were defined as the average of the difference within a simulation. Onset/offset jitters were defined as the standard deviation of the differences within a simulation. 

To determine the difference in performance between algorithms, we perform a pair-wise t-test by taking the difference in F1 scores between algorithms for each simulation. P-values were multiple-comparison corrected using the Bonferroni procedure.

### Experiment and Data

The York University Office of Research Ethics approved the present study as confirming to the standards of the Canadian Tri-Council Research Ethics guidelines (Certificate # 2016-214) We analyzed gaze data from performance during a feature-based rule-learning task (n=12) in a three-dimensional environment presented on a computer monitor, controlled using our laboratory’s publicly available USE software suite for active, video-game-like experiments (30). The task was similar to that described in the paper detailing this software (30), with the important differences that stimuli did not move, and responses were made using a combination of fixations and button presses instead of a joystick. We present the details of the task below, but note that since we simply required a large enough dataset of eyetracking data, the details are not relevant to the presented conclusions.

On each trial, participants selected one of two “Quaddle” objects (31), and received feedback on the accuracy of this choice. Through trial and error, they attempted to learn the rules governing reward. These objects had four feature dimensions (shape, surface colour, surface pattern, and arm type) with two different possible values each (i.e. the shape could be pyramidal or oblong, the colour could be red or orange, etc). Rules were always based on a single feature value, for example red objects might be rewarded and orange objects unrewarded, and each trial contained one rewarded and one unrewarded object. In each block there were two different rules, each operating in a different context (determined by the colour of the floor the objects were placed on). Once participants had made 10/12 correct choices, a new block began, and participants had to learn new randomly-selected rules.

At the beginning of each trial, participants were presented with a blank white screen and a fixation point that they needed to fixate for one second before they could begin a trial. Upon fixation, this central point would turn from blinking red to solid black, before disappearing. The blank white screen persisted for another 600ms before the subjects were presented with a 3D rendered arena with two objects placed at random positions. If participants broke this fixation too early, then the trial would be aborted.

Subjects had to fixate one of the two objects for 300 ms before receiving a cue that let them know that they were able to choose that object (the cue consisted of a translucent dot superimposed on stimulus). Participants were then able to choose that object by pressing down the spacebar and continuing to fixate the object for another 100 ms. Auditory feedback in the form of a low or high pitched beep, and visual feedback in the form of a red dot superimposed on the stimulus or a yellow dot superimposed on the stimulus for 300 ms, were used to indicate incorrect and correct responses respectively. After feedback, the objects disappeared, and an inter-trial interval consisting of the empty arena was displayed for 800ms. Participants had 30 seconds to make a response in a trial, otherwise the trial would be aborted, and they would be presented with instructions letting them know to respond faster.

The experiment was run using custom code for the Unity3D game engine. Gaze data was collected using a desktop mounted eyetracker situated well below eye-level (Tobii TX300; sampling frequency, 300 Hz), in a similar, low light environment. Participants were seated 50-60cm away from the monitor. Although they were seated, they were otherwise unrestrained.

We randomly selected 120 seconds of data from each participant at least 10 minutes into the session. Noise levels for each participant were defined as RMS of the x- and y-gaze positions during the inter-saccadic (i.e. fixational) periods (17). Manual classification was done with a custom GUI that had four displays; x-position, y-position, velocity, and (x,y) gaze. Classification was performed by four trained members of the lab with instructions to demarcate saccade and fixation onsets/offsets.

We compared algorithm performance as outlined above. To determine the effects of sampling rate, we resampled the data to a lower rate of 150 Hz (using the Matlab function *resample*). This was performed using the lambda for individual algorithms that gave the best over-all performance (lambda=6 for AT, lambda=9 for AT-MAD). We then determined if the difference in F1 scores significantly varied with the sampling rate.

To test for the effects of noise, we correlated subjects’ fixational noise (average of x and y RMS) with the difference in F1 scores.

## Results

To illustrate the strength of robust statistics, we show an example simulation with moderate noise where AT failed to detect the saccade (Figure 1C) but AT-MAD succeeded in doing so (Figure 1D; see also Figure 5E-F). In this example, the relatively high velocity values (Figure 1B) push the threshold higher than its initial starting value (Figure 1C). After many iterations, the threshold is too high to detect this saccade. However, because robust threshold estimation is relatively insensitive to outlier values, the AT-MAD algorithm successfully exits after one iteration, and is thus able to detect the saccade (Figure 1D). The average number of iterations across all algorithms and simulations ranged from 2 – 6, with robust algorithms exiting earlier than their non-robust counterparts at all noise levels.

**Figure 2.**
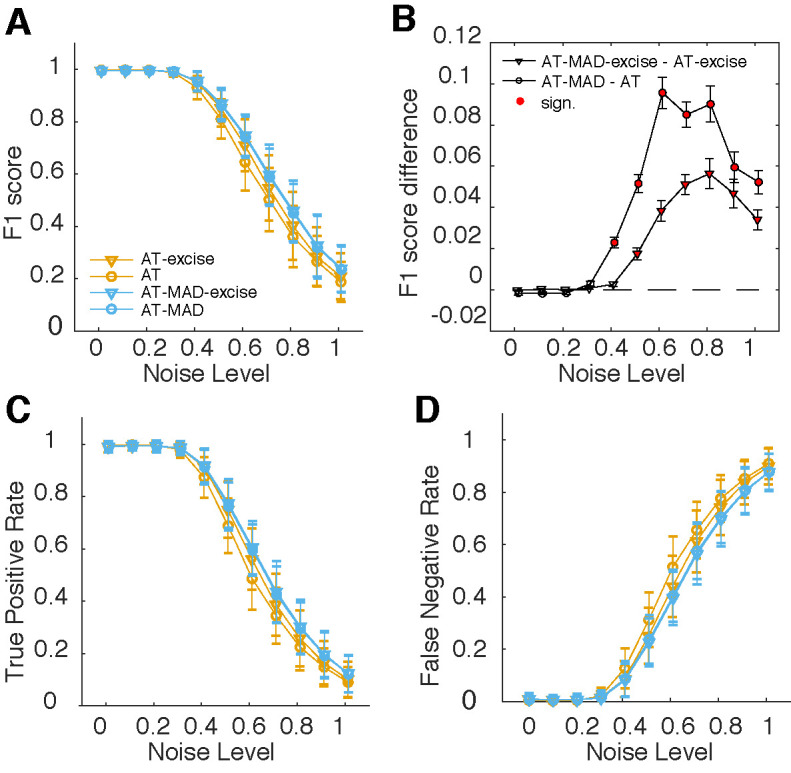
Robust estimation of threshold parameters results in improved detection performance **(A)** Mean and standard error of F1 score as a function of noise for two versions of the original algorithm (orange, AT and AT-excise) and two versions using the robust estimator (blue, AT-MAD and AT-MAD-excise). Versions using MAD consistently outperform (compare orange vs blue). Using the original data, excising some data improves detection performance (compare triangles and circles, orange lines), whereas it makes no difference for versions using the MAD estimator (triangles and circles, blue lines). **(B)** Mean and standard error of the pairwise difference in F1 score comparing AT vs AT-MAD (triangles), and AT-excise and AT-MAD-excise (circles). Filled, red circles represent statistically different score (p<0.05, multiple comparison corrected). Threshold estimation using MAD shows significantly improved performance for noise levels at 0.4 and above, particularly for the algorithm as originally proposed (triangles). **(C-D)** Mean and standard deviation of the true positive rate (C), and false negative rate (D) for the four different versions of the algorithm. Improvements in (A-B) can be traced both to a higher true positive rate (C) and a lower false negative rate (D).

We compared the detection performance (F1 score) of four different versions of the algorithm as a function of noise level (Figure 2A,B). We found that using the AT algorithm as originally proposed had the lowest performance. Performance improved if some data flanking the saccades was removed (AT-excise), as in the version published on Nyström’s website, suggesting that the original algorithm remains sensitive to the relatively high velocities just below threshold. However, AT-MAD and AT-MAD-excise had similar performance over all noise levels, showcasing the insensitivity of detected thresholds to outliers when using robust statistics. Robust threshold estimation consistently and significantly improved F1 scores by ~0.02-0.1 for noise levels equal to or greater than 0.4 (Figure 2B; p<0.05, multiple comparison corrected). If some data in the inter-saccadic intervals was excised (AT-excise vs AT-MAD-excise), the improvement peaked at 0.056, whereas the improvement was greatest (0.096) if all data was considered (AT vs AT-MAD). Improvements could be traced to both a higher true positive (Figure 2C) and a lower false negative rate (Figure 2D).

Previous studies have indicated that adaptive thresholds perform better than fixed thresholds (2, 11, 16), while other studies suggest that the inverse is true (8). To this end, we compared robust adaptive threshold estimation to a fixed threshold version of the algorithm (setting ${𝜃}_{\mathrm{PT}}=55$ , and ${𝜃}_{\mathrm{ST}}=45$ (8). We found that at low levels of noise (<0.2), fixed thresholds showed a minor advantage in performance (with a difference in F1 scores in the range of [0.0031, 0.0039]), but at high levels of noise (>0.3), adaptive threshold detection greatly outperformed fixed threshold detection (difference in F1 scores range, [0.24, 0.76]).

**Figure 3.**
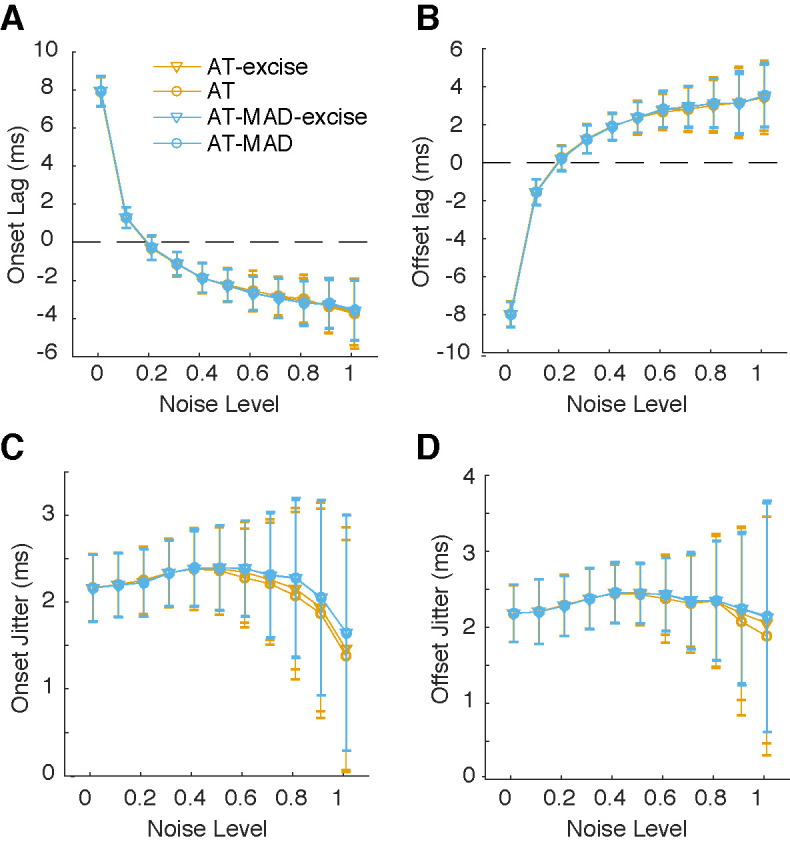
Onset and offset lags are comparable across all algorithms **(A-B)** Mean and standard error of the onset (A) and offset (B) lags, which is the difference in onset/offset as determined by the algorithm and the reference. All tested algorithms show similar lags. At higher noise levels, the lag decreases (shifted earlier). **(C-D)** Mean and standard deviation of the onset (C) and offset (D) jitter in lag. Across all tested noise levels, jitter is around 2 ms, but variability in jitter gets higher with increasing noise levels. Jitter at all noise levels is comparable across algorithms.

While robust threshold estimation results in better saccade detection, it may do so by failing to properly characterize saccade onsets and offsets. Thus, we compared onset and offset lags between the original and MAD versions of the algorithm. We found that onset and offset lags were comparable for all versions of the algorithms, increasing with noise (Figure 3A-B). All algorithms had a jitter of ~2ms across all noise levels, although the variability in jitter increased with noise (Figure 3C-D). Thus, based on the simulation results, the MAD algorithm consistently and more reliably detects saccades at higher noise levels, but shows similar saccade onsets/offsets.

**Figure 4.**
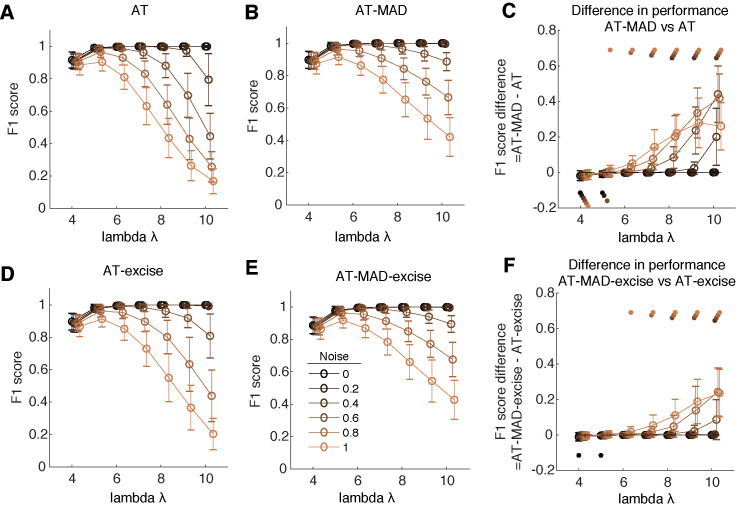
MAD is more robust to changes in threshold confidence level **(A)** Mean and standard deviation of the detection performance of the AT algorithm as a function of lambda for various levels of noise. Copper-tone color indicates the level of noise, with darker (lighter) colors indicating less (more) noise. At low noise levels (<0.2), performance does not depend on the choice of lambda. At moderate noise levels (0.4), performance rapidly decreases at very high lambda values (=10). However, with high noise levels, there is a substantial and rapid decrease in performance with higher lambda values. **(B)** Same as (A) but for the AT-MAD algorithm. Performance is high for low-moderate noise levels (<0.6). For higher noise levels, performance decreases at a slower rate for higher lambda values. **(C)** Difference in detection performance between AT-MAD and AT. Colored dots represent significant differences at the corresponding noise level. Dots above (below) zero depict significant increases (decreases) (p<0.05, multiple comparison corrected). At higher levels of lambda, AT-MAD far outperforms AT. This is especially true for moderate to high-levels of noise. **(D-F)** Same as (A-C) but for AT-excise (D), AT-MAD-excise (E), and their comparison (F). Results are qualitatively similar as for (A-C). Excising some data points using the original algorithm helps but using MAD still allows for higher lambda values

Experimental questions may necessitate varying levels of confidence in saccade detection. For example, studies comparing saccade characteristics across populations (4) may only wish to analyze well-defined saccades. In this case, experimentalists may wish to have a particularly high detection threshold. This can be controlled by setting the lambda parameter to higher values. To this end, we performed another set of analyses manipulating the value of lambda (Figure 4). We found that for low noise levels (<0.2), the value of lambda did not distinguish the performance of any four considered algorithms (Figure 4A-B,D-E). Very low lambda (=4, 5) had lower performance, due to a higher number of false positives. Here, AT-excise and AT slightly but significantly outperformed their robust counterparts (Figure 4C, F). However, the benefits of the MAD algorithm start to accrue at noise levels higher than 0.4. At these noise levels, very high lambda value (=10) negatively impact the performance of AT by a factor of ~0.2 (Figure 4A). As noise increases, performance of AT rapidly declined, to a minimum of ~0.2 at the highest noise level (=1). However, at this same level, AT-MAD achieved a performance of ~0.4. (Figure 4B). Indeed, for noise levels greater than or equal to 0.4, the AT-MAD algorithm had consistently and significantly higher performance than AT, up to a peak of ~0.45 at the highest value of lambda (Figure 4C). The effects were qualitatively similar when comparing AT-excise and AT-MAD-excise (Figure 4D-F), where the performance boost was smaller but still substantial (Figure 4F). Thus, the MAD algorithm allows experimentalists to more robustly define a desired confidence for detection.

**Figure 5.**
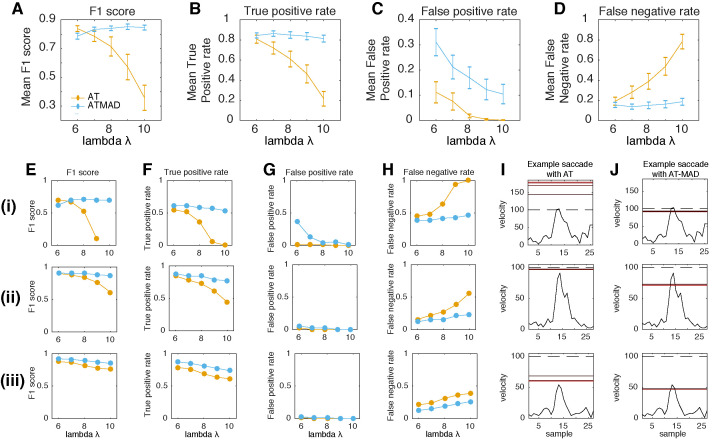
AT-MAD outperforms AT on real world data **(A-D)** Performance of the AT (orange) and AT-MAD (blue) algorithms on two minutes of human gaze data (n=12), depicted as the (A) F1 score, (B) True positive rate, (C) False positive rate, and (D) False negative rate. AT-MAD outperforms AT for all levels of lambda greater than 6. **(E-H)** Same as (A-D) but for three individual subjects (i-iii). In all three subjects, AT-MAD outperforms AT for lambda >6. In one subject (Ei), the F1 score is undefined because precision and recall are zero **(I,J) ** Example saccades in these subjects that AT could not detect (I) but AT-MAD could (J). Dashed line represents the initial threshold, and solid red lines are threshold on subsequent iteration, with darker (lighter) representing earlier (later) iterations.

 Up to this point, results were based on simulated data. A remaining question is how the algorithms would perform on real-world data. We analyzed data from twelve subjects performing a task with head-unrestrained viewing of a static scene. We manually annotated two minutes of data randomly sampled from each subject. Average noise levels (RMS) for the three subjects were 0.12 ± 0.015 dva in the horizontal direction, and 0.19 ± 0.024 dva in the vertical direction (32), placing it in the lower quarter of simulated noise levels. Noise levels were higher in the vertical rather than horizontal direction, as has been observed in adults using similar equipment (32), likely due to the pupil being occluded as it travels upwards and thus away from the eyetracking sensors. We then compared detection performance of the AT and AT-MAD (Figure 5). AT-MAD was equal to or outperformed AT in all cases where lambda was set to 7 or greater (Figure 5A). This was due to its higher true positive rate (Figure 5B), and lower false negative rate (Figure 5D). These improvements outweighed the higher false positive rate evident in AT-MAD rather than AT (Figure 5C). Three example subjects are shown in Figure E-J. Generally speaking, AT failed because it would consistently find a higher threshold than AT-MAD, resulting in an inability to detect low-velocity saccades (illustrated in Figure 5I-J). Detection performance of AT-MAD generally stayed stable across all values of lambda (Figure 5A), whereas AT showed a steep drop in performance with increasing lambda, as well as an increase in variance (Figure 5A). Indeed, in one subject, a lambda of 10 resulted in no detected saccades (Figure 5Ei). These results complement the simulation results and suggest that AT-MAD outperforms AT on human data by estimating a lower threshold, though one that is still conservative enough to avoid false positives in most cases. Furthermore, they suggest a lambda of at least 8 can drastically reduce false positives without affecting overall detection performance.

 Given these results, we focused our next analyses on the values of lambda that gave the best performance for each algorithm. This was a lambda of 6 for AT, and a lambda of 9 for AT-MAD. For these parameters, AT-MAD significantly outperformed AT (t-test, p=0.012), and found a lower threshold on average (AT: 94.6 ± 12.5, AT-MAD: 87.7 ± 9.68).

Our simulation results suggest that AT-MAD may be more suitable for noisier data. We thus related each subject’s average noise level (defined as the average of horizontal and vertical noise) with the difference in performance of AT-MAD and AT (where positive values indicate AT-MAD outperforms AT). The performance difference tended to be greater at higher levels of noise, though this did not reach significance (Spearman rank correlation, R=0.514, p=0.087). 

One potential difficulty in reliable saccade detection is the sampling rate of the acquisition equipment ((33). To this end, we compared the performance of the algorithms under lower sampling rates by down-sampling the data to 150 Hz. We then compared the performance difference under low (150 Hz, downsampled data) and moderate (300 Hz, original data) sampling rates across algorithms. We found that the performance difference between AT and AT-MAD was statistically indistinguishable (t-test, p=0.45).

## Discussion

The current work improves on saccade detection by robustly estimating a threshold while mitigating the influence of saccades themselves. The use of a robust estimator allows the estimation of a threshold unbiased by the very signal we are trying to detect (25). This allows a more accurate estimation of the background noise levels. We find that the use of MAD, a robust estimator of the dispersion not biased by outliers, improves the detection of saccades relative to previously published versions of this algorithm (11), particularly at higher noise levels. There is both an increase in the number of correctly identified saccades, and a decrease in the number of falsely identified saccades. Moreover, because MAD is not sensitive to outliers in the data, its use allows the experimentalist to confidently define their desired level of confidence.

The goal of the algorithm by Nyström and colleagues is to find the lowest possible threshold that can reliably differentiate saccades from noise and fixations. However, as we have shown, the resulting threshold remains sensitive to the saccades themselves. One reason is that data samples that fall just below threshold (i.e. those flanking detected saccades) can still influence the computed threshold. This concern can be alleviated by excising a number of samples in the inter-saccadic flanks. However, this procedure introduces another user-defined parameter, namely, the duration/number of samples to discard. It is likely the case that the optimal number of samples to discard varies by experimental condition, manipulation, or hard-ware considerations. By considering all of the data, this concern is obviated. 

We considered an experiment where the scene was static, and considered an appropriate algorithm for this. However, dynamic scenes present new challenges as they contain other gaze events, such as smooth pursuits. Smooth pursuits are difficult to distinguish from fixations and saccade events because of their overlapping velocity profiles, so an alternative is to consider the signal in the acceleration domain (7, 10, 22). Because the acceleration is a second order derivative, the effect of outliers is amplified. In this case, the use of the MAD estimator would likely improve threshold estimation, especially in combination with an iterative threshold estimation as Nyström and colleagues proposed (11). In fact, preliminary tests in our lab on other data where smooth pursuits are prevalent have shown this to be the case (data not shown). Other algorithms use thresholding on different aspects of gaze, such as dispersion, to disambiguate smooth pursuits from other gaze events (12, 34), and here too, we would expect robust threshold estimation to be beneficial during adaptive threshold estimation.

We have shown that the benefits of the MAD estimator mainly accrue at high noise levels. This is particularly important in non-ideal experimental conditions that can lead to noisier data, such as unrestrained viewing, or when working with younger, older, or clinical populations (4, 26), as well as in more realistic virtual or game-like settings (35). The other benefit of MAD is that it allows experimentalists to define a confidence level (lambda parameter), as required by the experimental questions/equipment. This could be relevant for studies that look at differences in saccade generation across individuals, or populations; such studies may wish to analyze only well-defined saccades (i.e. a higher confidence threshold) (4). Alternatively, lower thresholds may be used to allow the detection of micro-saccades (2), although this remains to be tested. The use of MAD allows experimentalists to set confidence levels in an unbiased manner.

The [XMLmind] study focuses on the comparison of one published algorithm with a robust alternative. This algorithm has laudable strengths compared to others, such as data-driven (as opposed to user driven) threshold estimation, and a flexible approach that allows setting different thresholds across different experimental subjects, sessions, trials etc. It performs better than nine published algorithms on experiments with static stimuli (12). That said, it remains an open question whether other threshold-based algorithms would benefit from the use of a robust estimator. Because of the ease of implementation, we believe this could be easily tested by interested readers.

This study is related to a method of threshold detection proposed by Engbert and colleagues (2), which has been widely used in research into micro-saccades (2, 3). They use a different, median-based method of estimating the standard deviation. A formal comparison between the two methods is beyond the purview of the paper. However, the present work should be seen as complementary. It provides a formal and rigorous comparison of robust vs non-robust statistics in the estimation of the standard deviation for the purpose of threshold determination and suggests that robust estimation may be superior in general. 

While the proposed method showed improvements in saccade detection, it did not affect the estimation of saccade onset and offsets. Thus, the use of robust statistics for threshold estimation should be viewed as complementary to methods that use more sophisticated approaches to determining saccade onsets and offsets that take into account gaze events such as post-saccadic oscillations, deviations from the main direction, or temporal changes in direction variability (e.g., Larsson et al., 2013). 

Our preliminary results suggest that both robust and non-robust saccade detection perform similarly at low (150 Hz) and moderate (300 Hz) sampling rates. However, low-frequency sampling is usually performed in experimentally challenging conditions (18, 36, 37), and thus this data tends to be noisier. Moreover, outliers exert more influence with less data. On the other hand, peak velocities cannot be reliably recovered under low-frequency sampling regimes (19), suggesting that the effect of outliers may be more prevalent at higher sampling rates. Future studies could ascertain the performance of robust and non-robust thresholding using different experimental equipment, including both commercially available and open-source systems (38). Additionally, robust estimation may be beneficial in non-human animal models, for which head-free eye-tracking is challenging. Indeed, initial testing in our laboratory suggests that robust threshold estimation improves saccade detection in non-human primates in a variety of different tasks (data not shown).

In conclusion, we present here a simple, easily implementable change to a common step in the analysis of saccades, namely, using a robust estimator of the central tendency and deviation to estimate detection thresholds. The simple change leads to improved saccade detection with a published algorithm. The simplicity of this change should encourage further testing and implementation in other thresholding algorithms.

### Ethics and Conflict of Interest

The author(s) declare(s) that the contents of the article are in agreement with the ethics described in http://biblio.unibe.ch/portale/elibrary/BOP/jemr/ethics.html and that there is no conflict of interest regarding the publication of this paper. 

### Acknowledgements

This work was supported by a grant from the Canadian Institute of Health Research CIHR Grant MOP_102482
